# Evaluation of the Ability of Diet-Tracking Mobile Applications to Estimate Energy and Nutrient Intake in Japan

**DOI:** 10.3390/nu12113327

**Published:** 2020-10-29

**Authors:** Nana Shinozaki, Kentaro Murakami

**Affiliations:** Department of Social and Preventive Epidemiology, School of Public Health, The University of Tokyo, 7-3-1 Hongo, Bunkyo-ku, Tokyo 113-0033, Japan; nana-s@m.u-tokyo.ac.jp

**Keywords:** mobile phone, smart phone, dietary record, dietary assessment, food composition database, portion size, Japan

## Abstract

We evaluated the energy and nutrient intake estimates of popular Japanese diet-tracking mobile applications (apps). We identified five diet-tracking apps in the iTunes store during August 2020. A researcher entered the dietary data from a one-day paper-based dietary record (DR) previously obtained from apparently healthy free-living adults (15 males and 15 females; 22–65 years) into each app. The energy and nutrient intakes estimated by the apps were compared with those calculated using the Standard Tables of Food Composition in Japan based on the paper-based DR (reference method). The number of dietary variables available ranged from one (energy in Mogutan) to 17 (FiNC). Compared to the DR-based estimates, the median energy intake was significantly overestimated by MyFitnessPal, Asken, Calomiru, and Mogutan. Moreover, the intakes of many nutrients were overestimated by Asken and Calomiru and underestimated by MyFitnessPal. For energy intake, the Spearman correlation coefficient between the DR and the apps was lowest for Mogutan (0.76) and highest for FiNC (0.96). The median correlation coefficient for nutrient intakes was lower in MyFitnessPal (0.50) than in the other three apps (0.80 in Asken, 0.87 in FiNC, and 0.88 in Calomiru). These results suggest that intake calculations differ among apps. Further evaluation is needed in free-living settings, where users input their own food intake.

## 1. Introduction

Dietary intake measurement is essential for nutritional epidemiological studies and clinical practices, including dietary counseling. Although traditional dietary assessments have relied on interviewer- or pen-and-paper-based methods, technological advances have resulted in innovative electronic approaches, such as web-based dietary records (DRs) [1,2,3]. Novel technology-based dietary assessment methods are considered to have the potential to reduce the burden and cost of data collection and to improve the validity and reliability of data [4,5,6,7].

In particular, with the widespread use of mobile phones, mobile applications (apps) that allow users can track their dietary intake are increasingly common [8,9,10]. Mobile phones have various technological features, such as wireless communication, portable design, and built-in cameras, making it possible to collect a wide range of information on people’s dietary intake and to provide feedback in real time [4,5]. In addition, people usually have higher satisfaction and preference for dietary assessment methods using mobile phones than traditional methods [4,11,12,13,14,15]. Thus, the use of diet-tracking mobile apps in nutrition research may be beneficial for both participants and researchers [16].

Since there are a substantial number of apps available, selecting a high-quality, fit-for-purpose tool is a major challenge [2,17]. Ideally, diet-tracking apps for research should have a user-friendly interface and experience, with an extensive and reliable food composition database (FCD) [3,16,18]. Most diet-tracking apps available in app stores (commercial apps) generally have user-friendly interfaces and a large volume of FCDs [16]. However, the accuracy of the nutrient intake calculation of these apps is not necessarily guaranteed due to the minimal governmental regulation of health apps [19]. To date, there have been only a few validation studies on consumer-oriented mobile apps, such as My Meal Mate and MyFitnessPal, both showing acceptable correlation with the reference methods [20,21]. Other evaluation studies comparing the accuracy of nutritional outputs from popular diet-tracking apps reported that the ability to estimate energy and nutrient intakes differed considerably between apps, with some apps providing inaccurate values [3,19,22,23,24,25]. Evaluation of diet-tracking apps would be helpful to understand their characteristics and potential utility in nutrition research while facilitating the evidence-based selection of tools and allowing for the development and improvement of these apps. To our knowledge, however, the accuracy of dietary intake estimated by commercial diet-tracking apps has not been extensively investigated. Furthermore, research has been limited to countries outside of Asia, such as the UK [3,20], the United States [19,25], Australia [14,24,26], the Netherlands [22], and Brazil [21,23].

This cross-sectional study aimed to evaluate the ability of diet-tracking mobile apps to estimate energy and nutrient intake in Japan. For the five most popular apps available in Japan, we entered food intake data previously obtained from a paper-based DR and compared the estimated dietary intakes from the apps with those calculated using the Standard Tables of Food Composition (STFCJ) in Japan based on the paper-based DR (reference method). Moreover, we investigated the energy and nutrient contents of four common foods provided by each app to assess the difference in these values in the FCDs of the apps.

## 2. Materials and Methods

### 2.1. Selection of Diet-Tracking Applications

**Figure 1:**
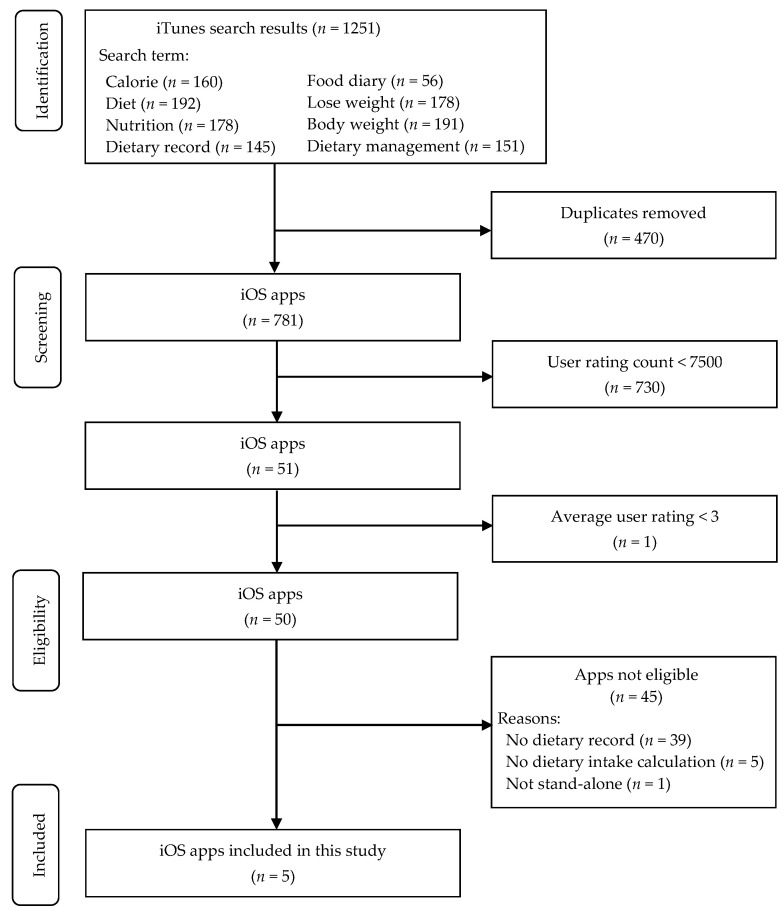
Flow diagram of app search and selection.

A systematic search for mobile diet-tracking apps was conducted in the Japanese iTunes store on 6 April 2020 (Figure 1). We searched iOS apps via the iTunes search app program interface [27], which allows the use of filters and search terms to explore content within the Apple iTunes store. The search was restricted to apps available in Japanese using the filter. Each of the following terms was separately inputted into a search query in Japanese: “calorie”, “diet”, “nutrition”, “dietary record”, “food diary”, “weight loss”, and “body weight.” These keywords were selected based on previous studies evaluating diet and nutritional apps [28,29,30,31]. The search results for each term were entered into the JavaScript Object Notation document, which was then converted and combined into an Excel file. This file includes the features of the apps, such as an app’s unique identifier, an app vendor’s URL, the app name, the description, the user rating, and the price [32]. Through this search process, we identified a total of 1251 apps. After removing duplicates (*n* = 470), 781 unique apps were screened for eligibility.

Apps were included in this study if they (1) were free of charge or freemium (i.e., free apps with limited functionality that is unlocked by purchasing the premium version); (2) had a food logging function; (3) had the ability to calculate energy or nutrient intake; (4) had a high star rating (≥4 out of 5) [31,33,34], (5) had at least 7500 reviewers [35]; (6) had standalone functionality (operating without other programs or equipment); and (7) were available in Japanese. If the information necessary to assess eligibility was not available in the app description, the apps were installed on an iPhone 8 (iOS 13.6) to obtain the details of app features. After excluding 776 apps, the following apps were considered eligible and included in this study: FiNC, MyFitnessPal, Asken, Calomiru, and Mogutan. All five apps were downloaded and installed on the iPhone for further evaluation.

### 2.2. Data Extraction

For each app, the following characteristics were extracted from the iTunes store, app vendor’s websites, and the content of each app: app name; vendor name; release date; content rating; average user rating and the number of ratings; price; language; connection with other devices; app function including passcode lock, reminders, and social networking options; and information collected about basic characteristics of users. Moreover, functions for dietary assessment were extracted, which included the following: food items available in the database, information source of the nutrient content of foods, category of eating occasions, assessment of the time of eating, input methods of food intake, methods to calculate nutrient intake, and output of dietary variables.

Furthermore, PubMed and Web of Science were searched to identify validation studies of the apps using a combination of each app name and the following string: (validity OR validation) AND (diet OR dietary OR intake OR consumption). We included studies that investigated the validity of estimated dietary intakes in comparison with a reference method such as DR. Studies were excluded if they used the website version of apps, which might differ from the mobile version, or if dietary data were manually entered by researchers into the apps. In addition, the references of the articles identified were also assessed to further identify relevant articles. We extracted the following information from eligible papers: first author, year of publication, country, participant characteristics (*n*, gender, and age), reference method, number of times an app was used, dietary intake variables analyzed, and the results of statistical tests.

### 2.3. Comparison of Dietary Intake between a Paper-Based Dietary Record and Applications

#### 2.3.1. Dietary Record Data

A four-day weighed DR was obtained from a nationwide survey conducted in 20 study areas consisting of 23 prefectures between February and March 2013, as described elsewhere [36,37]. Briefly, the study population consisted of apparently healthy adults aged 20–69 years working at welfare facilities as well as the family members or acquaintances for those over 60 years of age. The recruitment was conducted such that each study area included two males and two females from each of the 10-year age categories (20–29, 30–39, 40–49, 50–59, and 60–69 years), resulting in invitation of 400 participants. One individual per household could participate in the survey. None of the participants were a dietitian or a medical professional, received dietary therapy by a doctor or dietitian, had a history of educational hospitalization for diabetes mellitus, or was pregnant or lactating.

Participants were asked to record all foods and beverages consumed for four nonconsecutive days (three working days and one nonworking day, excluding days before and after a night shift). Research dietitians at each welfare facility explained to the participants how to keep the DR and requested them to weigh foods and beverages using a provided digital scale or measuring spoon and cup. The research dietitians collected and checked the record sheets soon after recording and, if necessary, asked participants further information to clarify the name or amount of food on the sheet. All food codes and weights were then reconfirmed by two other research dietitians at the central office of the study.

In total, 392 individuals (196 men and 196 women) completed the study protocol. Body weight (to the nearest 0.1 kg) in light clothes and body height (to the nearest 0.1 cm) without shoes were measured using standardized procedures by research dietitians or medical workers. Body mass index (BMI) was calculated as body weight (kg) divided by the square of body height (m^2^). The demographic characteristics of the study participants are provided elsewhere [37,38]. Energy and nutrient intakes for individuals were calculated based on the weight of food items and their nutrient content, using the STFCJ [39]. Dietary supplements were not included in the calculation of dietary intake. The study was conducted according to the guidelines in the Declaration of Helsinki, and all procedures were approved by the Ethics Committee at the University of Tokyo, Faculty of Medicine (No. 10005).

#### 2.3.2. Sample Size

The minimum sample size for assessing the difference in estimates between the DR and the apps was determined based on a previous study with a similar research question [3]. For two-tailed Wilcoxon signed-rank tests with a significance level of 0.05, power of 80%, and an effect size of 0.56, a sample size of at least 22 was required. Thus, we selected 30 participants through stratified randomization such that three males and three females from each of the five 10-year age categories were included. For each of these participants, the DR conducted on the first day over the recording period was used for data input in the apps, considering the change in dietary intake over the recording period [40].

#### 2.3.3. Input of Dietary Record Data into Applications

Food items on each participant’s DR, except for drinking water and dietary supplements, were entered into each of the five apps by the first author (registered dietitian) in a uniform procedure. For branded food products and restaurant menus, the databases in the apps were searched by the names of the brand, product, or restaurant, and then, the directly matched item was selected. If there was no match, a similar generic food or dish was selected. Generic single foods were searched by the food name; if there was no directly matched food, a biologically similar food was selected. Generic mixed dishes were searched by a dish name or main food ingredients, and a dish that best represented the cooking method or food ingredients was chosen. If there was no matched dish, a dish with similar main ingredients or cooking methods was selected. To investigate the agreement between each food item selected in the app and the original food item recorded in the DR, food items were categorized into “closely matched foods” or “poorly matched foods”. The former included food items that directly matched the items recorded in the DR, and the latter included items selected as similar foods.

The portion size of selected items in the app was adjusted for the best approximation of the net weight of that item recorded in the DR. For apps where the portion size needed to be entered as the ratio to a standard serving size (e.g., one serving), despite no information available on the standard weight, the portion size that seemed most appropriate was selected by considering the amount of food consumed. FiNC and Asken, in which several nutrients were unavailable in the free version, were upgraded to the premium version to obtain intake values. Estimated energy and nutrient intakes of each participant calculated by the apps were manually entered into Excel files, except for MyFitnessPal, which has a function for exporting data as CSV (Comma Separated Values) files.

### 2.4. Assessing the Energy and Nutrient Content of Food Items in the Database of Applications

To assess the energy and nutrient values of individual food items in the apps, four common food items were entered into each app. The energy estimates and macronutrient content (protein, total fat, and carbohydrate) were then compared with the reference values. The four items were selected from foods that existed in most of the apps and had a high consumption frequency in the 4-day DR from the entire study population (*n* = 392). The selected items were as follows: Pocky (chocolate-covered pretzel; Ezaki Glico Co., Ltd., Osaka, Japan) as an example of a branded food product, stewed chicken curry (Ichibanya Co., Ltd., Aichi, Japan) as an example of a restaurant meal, white rice as an example of a single generic food, and tonjiru (miso soup with pork and vegetables) as an example of a generic mixed dish.

If the apps showed more than one search result for each food item, a food that best represented each food and appeared at the top of the search results was selected. The reference values for Pocky and stewed chicken curry were obtained from nutrient information on the manufacturer’s websites, while those for white rice and tonjiru were obtained from the STFCJ [39]. Due to a lack of information on the portion size of mixed dishes (including tonjiru) in the STFCJ, the reference value for tonjiru was calculated as the nutrient content per 100 g of tonjiru in the STFCJ multiplied by the mean weight of the tonjiru dishes (311 g) recorded in the 4-day DR obtained from the entire study population.

### 2.5. Data Analysis

To describe each app, we tabulated the basic and nutrition-related features and the results of the validation studies. Moreover, the percentage of food items categorized into “closely matched foods” was calculated for each app. Energy and nutrient intakes estimated by each app were compared with those estimated from the DR using the STFCJ as a reference. Although trans fatty acid intake was calculated by MyFitnessPal, the STFCJ did not have the analytical value of trans fatty acids in foods. In addition, some apps offered to calculate sugar intake, while the type of sugar that the app estimated (total sugar, added sugar, or free sugar [41]) was not clear. Therefore, these two nutrients estimated from the apps were excluded from the comparison with the DR.

To assess the ability to estimate absolute intake among the apps, we compared mean and median intakes between the apps and the DR using paired *t*-test and Wilcoxon signed-rank test, respectively. Since both showed similar results, the results of the median comparison are shown here. Next, the ranking ability of the apps was evaluated using Spearman correlation coefficients with DR-based estimates. To summarize the correlation coefficients for each nutrient, we calculated the median value of the correlation coefficient of nutrients for each app. Additionally, a Bland–Altman plot [42,43] was used to assess the agreement of energy and macronutrient intakes (protein, total fat, and carbohydrate) estimated using the apps and those estimated by the DR. The upper and lower limits of agreement were calculated as the mean difference ± 1.96 standard deviations (SD). The proportional error between the two methods was evaluated using regression analysis. 

Finally, we counted the number of search results for four common food items and calculated the relative differences of energy and macronutrients estimated by the apps from the reference values. The relative difference was calculated as follows: relative difference (%) = ((app − reference)/reference) × 100. The statistical software package SAS version 9.4 was used for the analyses. Two-sided *p* values < 0.05 were considered statistically significant.

## 3. Results

### 3.1. Characteristics of the Selected Diet-Tracking Applications

There was some variation of features between the apps (Table S1). All the apps had a selection of functions available only for the paid version, except for the Mogutan app. Most apps were available only in one or two languages, except for MyFitnessPal, which was originally developed in the US and supported 20 languages. The app features related to dietary assessment are shown in Table 1. General foods and mixed dishes were included in the database of all the apps, whereas restaurant meals or branded food products were not covered in the Mogutan app. The number of food items in the database was largest in MyFitnessPal (*n* ≥ 4,000,000) and smallest in Mogutan (*n* = 278). For FCDs, MyFitnessPal, Asken, and Calomiru used their own estimated values and the nutrient data reported by food manufacturers. MyFitnessPal and Asken also use nutrient data from the national nutrient databases of the United States and Japan, respectively. Moreover, MyFitnessPal has a crowd-sourced database, making it possible for users to upload food entries. Foods are mainly selected using the text search of the databases, while items are registered by selecting food stickers in the Mogutan app. The amount of food consumed is adjusted by entering the percentage to the standard serving size in most apps, while it is adjusted by selecting a portion size from three categories in Mogutan. In addition, the amount of food can be directly entered into the MyFitnessPal app. The maximum number of dietary variables available, including the premium version, ranged from one (energy in Mogutan) to 17 (FiNC). Two validation studies [21,44] were identified for MyFitnessPal (Table S2). Compared to paper-based DRs or 24-h dietary recalls, underestimation of nutrients by the app was observed in both studies.

### 3.2. Comparison of Dietary Intake between the Applications and the Dietary Record

The percentage of food items classified as closely matched foods was the highest in the FiNC (95%) app, followed by MyFitnessPal (93%), Asken (92%), Calomiru (88%), and Mogutan (57%). Table 2 shows the median intakes of energy and nutrients estimated by the DR and those estimated by the five apps in the thirty participants (mean age, 44.5 (SD: 13.4, range: 22–65) years; mean BMI, 22.7 (SD: 3.0) kg/m^2^). Energy intake was significantly overestimated by MyFitnessPal, Asken, Calomiru, and Mogutan compared with that estimated by the DR. Among the apps providing nutrient intakes, FiNC had the lowest number of nutrients that significantly differed between the DR and the apps (only for niacin intake). MyFitnessPal significantly underestimated saturated fatty acids, monounsaturated fatty acids, polyunsaturated fatty acids, cholesterol, total dietary fiber, sodium, and potassium. On the other hand, Asken overestimated the intake of many nutrients, including carbohydrates, total dietary fiber, sodium, calcium, iron, and vitamin D. Similarly, Calomiru overestimated the intake of protein, carbohydrates, and sodium.

Spearman correlation coefficients for estimates between the DR and the apps are shown in Table 3. The correlation coefficient for energy was the lowest for Mogutan (0.76) and the highest for FiNC (0.96). Although the number of nutrients estimated differed among the apps, the median correlation coefficient for nutrients was lower in MyFitnessPal (0.50) than in the other three apps (0.80 in Asken, 0.87 in FiNC, and 0.88 in Calomiru).

**Figure 2:**
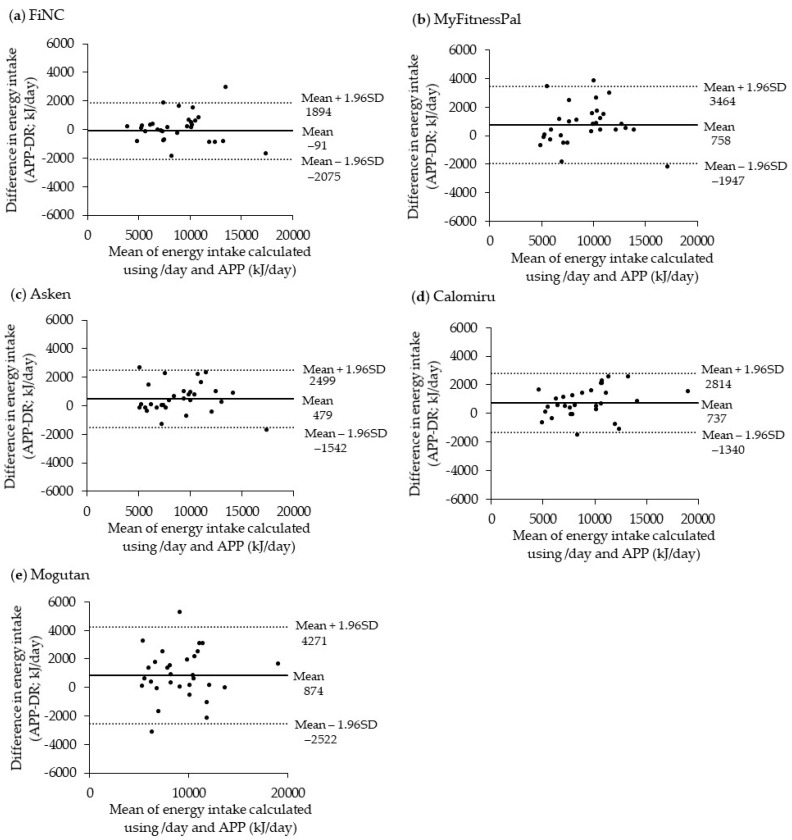
Bland–Altman plots assessing the agreement of the estimated energy intake between a paper-based dietary record (DR) and each application (APP) in 30 Japanese adults: (**a**) FiNC, (**b**) MyFitnessPal, (**c**) Asken, (**d**) Calomiru, and (**e**) Mogutan. The solid line represents the mean difference, and the dotted line represents lower and upper 95% limits of agreement.

Bland–Altman plots used to assess the agreement of intakes between the DR and the apps are shown in Figure 2 (for energy) and Figures S1–S3 (for protein, total fat, and carbohydrate, respectively). For energy, MyFitnessPal and Mogutan had greater mean differences and wider limits of agreements. For all the macronutrients (plotted except for Mogutan), the mean differences were the largest in Calomiru and the limits of agreement were the widest in MyFitnessPal. Regression analysis showed significant linear trends in protein intake estimated by FiNC (β = 0.19, *p* = 0.01) and Calomiru (β = 0.38, *p* = 0.001).

### 3.3. Energy and Nutrient Content of Four Food Items

The energy and nutrient contents of the four food items are shown in Table 4. The number of search results was the largest in MyFitnessPal for three of the four items. Moreover, Mogutan showed large differences in energy content from the reference value for all items. For Pocky and stewed chicken curry, the energy and macronutrient contents in FiNC and Asken were not consistent with the reference value. For white rice, MyFitnessPal overestimated energy and nutrient contents, up to a maximum of 200% difference (total fat). FiNC showed 0–4% relative differences in energy and nutrient contents, since the amount of rice could not be adjusted to 100 g using FiNC, which employed portion size adjustments in units of 10%. The energy and nutrient content of tonjiru was overestimated by all the apps, with a range of 61–264%.

## 4. Discussion

### 4.1. Summary of Results

In this study, we described the features of five popular diet-tracking mobile apps in Japan and evaluated their ability to estimate energy and nutrient intake. Consistent with previous studies [3,19,22,23,24,25], there were vast differences in estimated intakes among the apps. FiNC could adequately estimate energy and nutrient intake both at the group and individual levels and could rank individuals according to their intakes.

In comparison, these features were poor in MyFitnessPal and Mogutan. Although Asken and Calomiru had moderate ranking capabilities, they demonstrated inadequacy regarding the estimation of energy and nutrient intakes at the group level. Moreover, Calomiru was associated with a poor ability to estimate energy and nutrient intakes at the individual level. This may be attributable to the difference in the functions of apps relevant to nutrient intake calculation, such as FCDs used for estimating dietary intakes, methods to search and select food items, and portion size estimation. Moreover, only MyFitnessPal had been previously validated, indicating the lack of scientific evidence in most of the apps. To our knowledge, this is the first study to evaluate the ability of commercial diet-tracking mobile apps to estimate energy and nutrient intake in Japan.

### 4.2. Food Composition Databases

The extensiveness of food selections in FCDs may affect the efficacy of users in the selection of food items that exactly match the foods they consume. The number of food items in the database was the smallest in Mogutan, which did not include restaurant or brand-name food products. Consequently, Mogutan had the lowest percentage of food items that matched food items in the DR, resulting in reduced ability to estimate energy intake compared to other apps. On the other hand, MyFitnessPal, which used the FCD of the US and a crowd-sourced database, had the greatest number of food selections, including restaurant and brand-name foods, and a high percentage of food items matching foods in the DR. Nevertheless, MyFitnessPal had a poor ability to estimate energy and nutrient intake both at the group and individual levels. This paradox may be due to the fact that the US-based FCD may differ in food selections and nutrient content in foods when compared to Japanese databases [25]. Moreover, although the crowd-sourced database is potentially beneficial for enriching the data, given that users can upload food items by entering only the food name and energy content, some nutrient values may be incorrect or missing, especially for foods that are not labeled [3,16,19,21,22]. Indeed, MyFitnessPal underestimated the intake of nutrients that are not required for food labels in Japan [46], including fatty acids, cholesterol, total dietary fiber, sodium, and potassium. The underestimation of nutrients by MyFitnessPal was also reported in two validation studies [21,44]. Although users can correct inaccurate values, the efficacy of such user-based quality control remains unclear [22].

Although FiNC and Asken used the manufacturer’s data for the FCDs, we found that the energy and nutrient values of two commercial foods (Pocky and stewed chicken curry) were not consistent with the reference values. Since food products are reformulated by manufacturers, nutrient values in the apps may be outdated [25]. The continuous update of large food product databases remains a key challenge for diet-tracking apps [2,22]. Furthermore, the energy and nutrient contents of tonjiru were considerably overestimated in all the apps, indicating the difficulty in estimating the nutrient content of mixed dishes. Given that the Japanese diet consists primarily of a variety of mixed dishes [47], the misestimation of energy and nutrient intake by the apps may be derived mainly from the difference in the nutrient content of mixed dishes between the FCD of apps and those actually consumed.

The FiNC, Calomiru, and Mogutan apps did not provide all the information sources of nutrient values. Moreover, although some apps calculated sugar intake, the type of sugar that the app estimated was unclear. As a result, sugar intake provided by the apps differed considerably. Previous studies have also reported that information on the development process of apps or the source of nutrient values was not available for many commercial apps [7,23,30]. The lack of transparency in the information may hinder the use of diet-tracking apps in nutritional research and practice.

### 4.3. Portion Size Estimation

Portion size estimation is important since misreporting of the consumed amount is a major source of measurement error when assessing dietary intake [44,48,49,50]. In this study, all apps estimated the amount of food consumed based on the relative portion size entered by users. However, methods to record the relative portion size differed among apps. While most apps allowed minor adjustments of the relative portion, Mogutan offered only three categories of food portion size and FiNC required entry of the relative portion size in a unit of 10%. Therefore, the exact amount of food could not always be entered in these apps. However, since the FiNC showed the standard weight for all food items, the best approximation of the relative portion size was entered based on the grams of foods written in the DR. This may explain the agreement of estimates in FiNC with the DR. Meanwhile, the other apps did not show the weight or picture of the standard portion for most food items, making it difficult to estimate the relative portion size. Noticeably, MyFitnessPal had the option to enter the quantity of foods based on units of grams or milliliters. Although accurate recording of the food amount appears to be useful, entering the precise amount of food or beverages may not always be easy, at least for some users [21,44]. Therefore, showing portion size images or household measurement units for each food item would help improve portion size estimation [44].

### 4.4. Other Features

Other app features related to usability may affect how accurately users input their food intake into apps. For instance, as the Japanese language is composed of three types of characters (hiragana, katakana, and kanji), searching for food names by different spellings produced different results in most apps. In addition, since MyFitnessPal supports 20 languages, searches in Japanese sometimes resulted in foods written in other languages, such as Chinese and English. This may make it difficult to find an exact food from a large database. Moreover, although users generally prefer large food databases, identifying correct foods from numerous food choices is complicated [44,51]. Therefore, easy-to-navigate food databases, in which the number of food entries is not overwhelming, are needed to facilitate accurate food selection [18]. In any case, to use these apps in research, more validation studies in a free-living setting are needed.

### 4.5. Limitations

Several methodological limitations of this study warrant mention. First, because our search was restricted to popular apps available for free in the Apple iTunes store, there may have been other relevant paid apps or apps distributed at other stores such as Google Play [34]. Our search strategy was based on the fact that free apps are widely used with many downloads [25,52], that the iPhone has 59.8% of the market share in Japan in 2019 [53], and that the Apple store and Google play store offerings overlap [19]. Second, we did not evaluate the actual use of apps in a free-living setting. Since dietary data were entered into the app by the author, who was a registered dietitian, the selection of foods may be more accurate compared to general users. In addition, we entered the amount of foods and beverages based on the weighed amount recorded in the DR, whereas general users may be more likely to enter portion size based on a rough estimate rather than based on weighing the amount of foods or beverages. Furthermore, the accuracy of data input may also be influenced by personal characteristics of users, such as technology literacy and personal preference [51]. Given that this study design in theory eliminated these biases involved with user entry, the concordance of estimates between the apps and DR may have been overestimated. Therefore, validation studies among free-living users are required to investigate the accuracy of estimates from apps more rigorously. Third, the input of dietary data was conducted by a single researcher and was not verified by others due to the limited number of researchers. This may have resulted in input errors and eventually misestimation of the apps’ ability. The input of dietary data by more than one person independently would be desirable for a more rigorous evaluation. Fourth, dietary data used were collected from healthy volunteers who were not randomly selected. Moreover, we assessed only thirty dietary data records, mainly due to time constraint, although the sample size was calculated based on a previous study. Therefore, the selection of foods or the amount may not be representative, which may have affected the results of the evaluation of each app. Hence, although the sample size was sufficient to examine our research question, the results should be confirmed based on a larger number of data entries to increase the reliability of the findings. Finally, we did not evaluate the accuracy of estimates provided by the barcode scanner or automated image analysis of apps. The evaluation of these technologies to make food entry easier and quicker would be of interest in further studies.

## 5. Conclusions

In conclusion, this study investigated the features and abilities to assess dietary intake among the five popular diet-tracking apps in Japan. Each app varied in the ability to estimate nutrient intake among apps, which may be explained by the difference in app features, such as FCDs and the method to input the information regarding food intake. Although estimates from some apps were comparable to those from the paper-based DR, the results should be interpreted with caution due to the methodological limitations of this study. This study contributes to a better understanding of the ability of diet-tracking mobile apps for clinicians and researchers as well as an improved development of these types of apps. The finding reinforces the need for evaluation of diet-tracking apps to explore their potential use in nutrition research and practice. In particular, validation studies where users input their own food intake in free-living settings are needed to evaluate the ability of dietary-tracking apps more rigorously.

## Figures and Tables

**Table 1:** Nutritional functionalities of the five diet-tracking applications included in this study ^a^.

Function	FiNC	MyFitnessPal	Asken	Calomiru	Mogutan
Food items available in the database					
General foods and dishes	✓	✓	✓	✓	✓
Restaurant meals	✓	✓	✓	✓	None
Branded food products	✓	✓	✓	✓	Only four items
Number of food items	No information	≥4,000,000	≥100,000	About 4000 for general foods/about 15,000 for restaurant meals and branded food products	278
Sources of nutrient content of foods					
Food manufacturers	No information	✓	✓	✓	No information
Estimation by application vendors	No information	✓	✓	✓	No information
National nutrient databases	No information	USDA SR Crowd-sourced database ^b^	STFCJ 2015	Not specified ^c^	No information
User-generated data	None	✓	None	None	None
Input of eating occasion					
Eating occasion category	Breakfast/lunch/dinner/snack	Customizable up to six categories	Breakfast/lunch/dinner/snack	Breakfast/lunch/dinner/snack	Breakfast/lunch/dinner/snack
Time of eating	✓	✓	✓	✓	None
Input methods of food intake					
Food images	✓	None	✓ ^d^	✓	None
Text search from food databases	✓	✓	✓	✓	None
Barcode scanner	None	✓	None	None	None
Original recipes or foods	None	✓	None	✓	✓
Other	None	None	None	None	Select from food stickers
Quantification of food intake	Percentage to standard serving sizes (unit: 10%)	Percentage to standard serving sizes (unit: 1%) or amount (gram/milliliter/cup/ounce)	Percentage to standard serving sizes (unit: any percentages) or energy content	Percentage to standard serving sizes (unit: 1%)	Three PS categories: all/half/a little or energy content
Methods to calculate nutrient intake					
Manual calculation by dietitians	None	None	None	✓	None
Automated calculation from inputted food intake	✓	✓	✓	✓	✓
Semiautomatic image analysis	✓	None	None	✓	None
Output of dietary variables (shown as intake values)	Energy and 15 nutrients (+sugar for the premium version)	Energy and 12 nutrients	Energy (+13 nutrients for the premium version)	Energy and 5 nutrients	Energy
Validation studies	None	Two studies	None	None	None

PS, portion size; STFCJ, Standard Tables of Food Composition in Japan; USDA SR, US Department of Agriculture National Nutrient Database for Standard Reference. ^a^ Functions are for the free version of each app unless otherwise indicated. The check mark represents that apps have the respective features. ^b^ Users can freely upload food items and correct the energy and nutrient content. ^c^ Referred to as “food composition databases” (no further information). ^d^ Users can register food photos as memos although the photos are not analyzed.

**Table 2:** Median intakes of energy and nutrients estimated by a one-day paper-based dietary record (DR) and those estimated by the five diet-tracking applications among Japanese adults (*n* = 30).

	Paper-based DR ^a^			FiNC ^b,c^				MyFitnessPal ^b^				Asken ^b,d^				Calomiru ^b^				Mogutan ^b^			
Variables	Median	*P*_25_	*P*_75_	Median	*P*_25_	*P*_75_	*P* ^e^	Median	*P*_25_	*P*_75_	*P* ^e^	Median	*P*_25_	*P*_75_	*P* ^e^	Median	*P*_25_	*P*_75_	*P* ^e^	Median	P_25_	P_75_	*P* ^e^
Energy (kJ/day)	8556	6514	10,095	8512	6853	10,887	0.77	10,170	6936	11784	0.003	9065	6732	11,891	0.02	9010	7376	11,803	0.001	9525	7092	11,724	0.004
Protein (g/day)	69.2	61.7	80.9	72.5	56.0	83.0	0.83	65.3	53.3	86.2	0.88	75.2	56.6	97.9	0.06	74.8	62.6	100.0	0.0004	-	-	-	-
Total fat (g/day)	59.1	34.2	74.8	58.0	39.0	88.0	0.33	56.1	39.9	79.9	0.46	61.0	42.6	80.4	0.09	60.7	38.3	85.9	0.06	-	-	-	-
Saturated fatty acid (g/day)	14.6	9.6	23.3	-	-	-	-	2.1	0.4	6.0	<0.0001	17.1	11.0	23.8	0.99	-	-	-	-	-	-	-	-
Monounsaturated fatty acid (g/day)	20.9	11.9	27.8	-	-	-	-	2.7	0.0	4.7	<0.0001	-	-	-	-	-	-	-	-	-	-	-	-
Polyunsaturated fatty acid (g/day)	10.9	7.7	14.3	-	-	-	-	1.6	0.4	3.3	<0.0001	-	-	-	-	-	-	-	-	-	-	-	-
Trans fatty acid (g/day)	-	-	-	-	-	-	-	0.0	0.0	0.0	-	-	-	-	-	-	-	-	-	-	-	-	-
Cholesterol (mg/day)	338	219	549	-	-	-	-	8	0	23	<0.0001	-	-	-	-	-	-	-	-	-	-	-	-
Carbohydrate (g/day)	274.3	207.5	338.0	267.5	209.0	342.0	0.74	282.3	192.7	345.8	0.45	292.4	207.1	346.9	0.03	304.7	202.8	354.7	0.008	-	-	-	-
Sugar ^f^ (g/day)	-	-	-	254.5	199.0	326.0	-	5.5	0.0	16.9	-	-	-	-	-	291.2	196.2	342.6	-	-	-	-	-
Total dietary fiber (g/day)	12.7	8.4	16.2	12.5	9.0	16.0	0.24	6.8	3.1	10.5	0.0002	20.5	10.6	25.5	<0.0001	13.1	9.4	16.8	0.10	-	-	-	-
Sodium ^g^ (mg/day)	3994	2700	4644	3780	2953	4803	0.64	2893	2039	4215	0.03	4213	3346	5591	0.004	4750	3839	5264	<0.0001	-	-	-	-
Potassium (mg/day)	2484	2032	2981	2501	2026	2945	0.46	1033	331	1580	<0.0001	-	-	-	-	-	-	-	-	-	-	-	-
Calcium (mg/day)	447	312	664	437	320	720	0.46	- ^h^	-	-	-	501	389	640	0.002	-	-	-	-	-	-	-	-
Magnesium (mg/day)	270	227	314	274	214	330	0.45	-	-	-	-	-	-	-	-	-	-	-	-	-	-	-	-
Iron (mg/day)	7.3	5.6	8.8	7.4	6.0	9.3	0.20	- ^h^	-	-	-	8.2	5.9	10.2	0.03	-	-	-	-	-	-	-	-
Vitamin A ^i^ (µg/day)	391	255	628	-	-	-	-	- ^h^	-	-	-	450	220	807	0.07	-	-	-	-	-	-	-	-
Vitamin D (µg/day)	4.0	1.7	8.4	2.6	1.5	5.6	0.07	-	-	-	-	-	-	-	-	-	-	-	-	-	-	-	-
α-Tocopherol (mg/day)	6.4	4.0	8.3	-	-	-	-	-	-	-	-	8.6	5.6	11.0	0.0001	-	-	-	-	-	-	-	-
Thiamin (mg/day)	0.95	0.64	1.32	1.00	0.70	1.60	0.10	-	-	-	-	1.03	0.64	1.33	0.23	-	-	-	-	-	-	-	-
Riboflavin (mg/day)	1.24	1.02	1.51	1.40	0.90	1.70	0.33	-	-	-	-	1.42	0.97	1.66	0.86	-	-	-	-	-	-	-	-
Niacin (mg/day)	17.7	13.0	21.5	32.5	23.0	39.0	<0.0001	-	-	-	-	-	-	-	-	-	-	-	-	-	-	-	-
Vitamin B-12 (µg/day)	3.3	2.6	7.0	4.9	2.9	9.0	0.06	-	-	-	-	-	-	-	-	-	-	-	-	-	-	-	-
Vitamin C (mg/day)	81	44	121	94	48	130	0.35	- ^h^	-	-	-	90	49	125	0.27	-	-	-	-	-	-	-	-

DR, dietary record; *P*_25_, 25th percentile; *P*_75_, 75th percentile. ^a^ Estimated based on a one-day paper-based DR using the Standard Tables of Food Composition in Japan [39]. ^b^ Estimated from the apps by entering the same food items on the DR (except for dietary supplements and drinking water) into each app. ^c^ Sugar intake is available for the premium version only. ^d^ Intake values of all nutrients (excluding energy) were available only for the premium version. ^e^ The difference from values derived from the DR was tested by the Wilcoxon signed-rank test. ^f^ The type of sugar that the app estimated was not clearly defined for FiNC or MyFitnessPal. For Calomiru, sugar intake is basically calculated as carbohydrate intake (g) minus the dietary fiber intake (g). ^g^ For comparison, salt intakes estimated by FiNC, Asken, and Calomiru were converted to sodium intake as follows: sodium (mg) = salt (mg)/2.54 × 1000 [45]. ^h^ Only the percentages to the dietary reference intakes of the dietary guidelines for Americans are shown in MyFitnessPal. ^i^ Retinol activity equivalent.

**Table 3:** Spearman correlation coefficients for the association between energy and nutrient intakes estimated by a one-day dietary record and those estimated by the five diet-tracking applications among Japanese adults (*n* = 30) ^a^.

	FiNC	MyFitnessPal	Asken	Calomiru	Mogutan
Variables	*r*	*r*	*r*	*r*	*r*
Energy (kJ/day)	0.96	0.90	0.95	0.93	0.76
Protein (g/day)	0.93	0.74	0.84	0.88	-
Total fat (g/day)	0.87	0.81	0.89	0.88	-
Saturated fatty acid (g/day)	-	0.26	0.86	-	-
Monounsaturated fatty acid (g/day)	-	0.33	-	-	-
Polyunsaturated fatty acid (g/day)	-	0.49	-	-	-
Cholesterol (mg/day)	-	0.23	-	-	-
Carbohydrate (g/day)	0.95	0.82	0.95	0.92	-
Total dietary fiber (g/day)	0.93	0.55	0.89	0.85	-
Sodium (mg/day)	0.81	0.47	0.73	0.76	-
Potassium (mg/day)	0.92	0.51	-	-	-
Calcium (mg/day)	0.92	-	0.84	-	-
Magnesium (mg/day)	0.91	-	-	-	-
Iron (mg/day)	0.85	-	0.80	-	-
Vitamin A ^b^ (μg/day)	-	-	0.65	-	-
Vitamin D (μg/day)	0.92	-	-	-	-
α-Tocopherol (mg/day)	-	-	0.76	-	-
Thiamin (mg/day)	0.67	-	0.72	-	-
Riboflavin (mg/day)	0.71	-	0.55	-	-
Niacin (mg/day)	0.82	-	-	-	-
Vitamin B-12 (μg/day)	0.70	-	-	-	-
Vitamin C (mg/day)	0.73	-	0.75	-	-

^a^ All correlation coefficients were significantly higher than 0 (*p* < 0.05) except for that for saturated fatty acid (*p* = 0.17), monounsaturated fatty acid (*p* = 0.08), and cholesterol (*p* = 0.21) in MyFitnessPal. ^b^ Retinol activity equivalent.

**Table 4:** Examples of energy and nutrient contents in the diet-tracking applications for four food items ^a^.

			Dietary Tracking Applications									
			FiNC		MyFitnessPal		Asken		Calomiru		Mogutan	
Food items		Reference ^b^	Value	Relative Difference (%) ^c^	Value	Relative Difference (%) ^c^	Value	Relative Difference (%) ^c^	Value	Relative Difference (%) ^c^	Value	Relative Difference (%) ^c^
**Pocky Chocolate, Glico, 1 pack**												
	Number of search results ^d^	-	2	-	>500	-	70	-	3	-	NA	-
	Energy (kJ)	761	732	−4	761	0	732	−4	761	0	318	−58
	Protein (g)	3.0	3.1	3	3.0	0	3.1	3	3.1	3	-	-
	Total Fat (g)	8.2	7.6	−7	8.2	0	7.6	−7	8.2	0	-	-
	Carbohydrate (g)	24.0	23.5	−2	24.0	0	23.5	−2	23.9	0	-	-
**Stewed chicken curry (pork source), CURRY HOUSE CoCo ICHIBANYA, 1 serving**												
	Number of search results ^e^	-	2	-	22	-	3	-	2	-	NA	-
	Energy (kJ)	3661	3561	−3	3661	0	3561	−3	3661	0	2720 ^f^	−26 ^f^
	Protein (g)	22.7	24.2	6	22.7	0	24.2	7	22.7	0	-	-
	Total Fat (g)	28.3	25.2	−11	28.3	0	25.2	−11	28.3	0	-	-
	Carbohydrate (g)	126.7	125.4	−1	126.7	0	125.4	−1	126.7	0	-	-
**White rice, cooked, 100 g**												
	Number of search results ^g^	-	35	-	432	-	26	-	>500	-	NA	-
	Energy (kJ)	703	728 ^h^	4	1063	51	703	0	698	-1	983	40
	Protein (g)	2.5	2.6 ^h^	4	6.1	144	2.5	0	2.5	0	-	-
	Total Fat (g)	0.3	0.3 ^h^	0	0.9	200	0.3	0	0.3	0	-	-
	Carbohydrate (g)	37.1	38.6 ^h^	4	77.1	108	37.1	0	37.1	0	-	-
**Tonjiru (miso soup with pork and vegetables), 1 serving**												
	Number of search results ^i^	-	163	-	>500	-	104	-	237	-	NA	-
	Energy (kJ)	351	849	142	1100	213	611	74	841	139	1059	201
	Protein (g)	4.7	10.6	127	12.6	170	9.1	95	9.4	102	-	-
	Total Fat (g)	4.7	11.9	155	17.0	264	7.5	61	12.6	170	-	-
	Carbohydrate (g)	6.2	12.7	104	15.2	144	10.3	66	11.9	91	-	-

NA, not applicable. ^a^ Energy and nutrient contents are shown for one food item that best represented each food in the search results in each app. ^b^ The reference values for Pocky and stewed chicken curry were obtained from nutrient information on the manufacturer’s websites. The reference values for white rice were derived from food code 1088, “rice, short grain, paddy rice, nonglutinous rice, well-milled, meshi (cooked rice)” in the Standard Tables of Food Composition in Japan (STFCJ) [39]. The reference values for tonjiru were calculated as the nutrient content per 100 g of tonjiru in the STFCJ multiplied by the mean weight of 313 tonjiru dishes (311 g) recorded in the 4-day DR obtained from the entire study population (n 392). ^c^ Relative difference (%) = ((app − reference)/reference) × 100. ^d^ Apps were searched using the keywords “Pocky chocolate” and “Glico”. ^e^ Apps were searched using the keywords “chicken nikomi curry” (menu name in Japanese) and “CoCo ICHIBANYA”. ^f^ The same product was not identified. Instead, the energy content of generic curry rice was shown. ^g^ Apps were searched using the keyword “hakumai” (white rice in Japanese). ^h^ The energy and nutrient values were shown for 104 g of white rice because the position size could not be adjusted to 100 g. ^i^ Apps were searched using the keywords “tonjiru” and “butajiru”.

## Supplementary Materials

The following are available online at https://www.mdpi.com/2072-6643/12/11/3327/s1, Figure S1: Bland–Altman plots assessing the agreement of the estimated protein intake between a paper-based dietary record (DR) and each application (APP) in 30 Japanese adults, Figure S2: Bland–Altman plots assessing the agreement of the estimated fat intake between a paper-based dietary record (DR) and each application (APP) in 30 Japanese adults, Figure S3: Bland–Altman plots assessing the agreement of the estimated carbohydrate intake between a paper-based dietary record (DR) and each application (APP) in 30 Japanese adults, Table S1: Basic information of the five diet-tracking applications included in this study, Table S2: Characteristics of the two validation studies for MyFitnessPal.
