# Supplementary material for: Evaluation of the Ability of Diet-Tracking Mobile Applications to Estimate Energy and Nutrient Intake in Japan
Source: Nutrients. 2020 Oct 29;12(11):3327. doi: 10.3390/nu12113327 (PMC7694045; doi:10.3390/nu12113327)
Supplement: Supplementary file 1 [file nutrients-12-03327-s001.pdf]

Table S1. Basic information of the five diet-tracking applications included in this study

Features	FiNC	MyFitnessPal	Asken	Calomiru	Mogutan
Vendor name (country)	FINC Technologies Inc. (Japan)	Under Armour, Inc. (US)	Asken, Inc. (Japan)	Life Log Technology, Inc. (Japan)	Mediano Co., Ltd. (Japan)
Release date	February 2015	December 2009	September 2013	February 2015	July 2014
Content rating	12+	4+	4+	4+	4+
User rating					
Average (range: 1—5)	4.13	4.43	4.25	4.34	4.16
Number of user ratings	39430	12148	28996	7878	10054
Price	Freemium [a]	Freemium [a]	Freemium [a]	Freemium [a]	Free
Language	Japanese/English	20 languages including Japanese [b]	Japanese/English	Japanese	Japanese
Connection with other devices	Wearable tracker, body composition scales, healthcare app	Wearable tracker, body composition scales, healthcare apps, fitness apps	Wearable tracker, healthcare app, fitness app	Wearable tracker, body composition scales, healthcare apps, fitness apps	None
Basic app functions					
Physical activity log	✓	✓	✓	✓	✓
Sleep diary	✓	None	✓	None	None
Password/passcode lock	✓	✓	✓	None	✓
GPS	✓	None	None	None	None
Educational information	✓	None	✓	✓	✓
Text message feedback	✓	✓	✓	✓	None
Social networking option	✓	✓	✓	✓	✓
Reminders	✓	✓	✓	None	✓
User incentive	✓	None	None	✓	✓
Customer support	✓	✓	✓	✓	✓
Advertisement	✓	✓	✓	✓	✓
Terms of service	✓	✓	✓	✓	✓
Privacy policy	✓	✓	✓	✓	✓
Data export	None	For premium only	None	None	None
Basic information collected					
Gender	✓	✓	✓	✓	✓
Date of birth/age	✓	✓	✓	✓	✓
Residential area/postal code	✓	✓	None	✓	None
Height and weight	✓	✓	✓	✓	✓
Body fat	✓	None	✓	✓	None
Neck circumference	None	✓	None	None	None
Waist circumference	None	✓	None	None	None
Hip circumference	None	✓	None	None	None
Target weight	✓	✓	✓	✓	✓
Physical activity level	None	✓	None	✓	None
Calculation of BMI	✓	None	None	None	None

App, application; GPS, Global Positioning System. [a] Free app with limited functionality which is unlocked by purchasing the full version. The check mark represents that apps have the respective features. [b] Japanese, Italian, Indonesian, Dutch, Swedish, Spanish, Danish, Turkish, German, Norwegian (Bokmål), Filipino, French, Portuguese, Polish, Malay, Russian, Simplified Chinese, Traditional Chinese, English, and Korean.

Table S2. Characteristics of the two validation studies for MyFitnessPal

First author (year) country	Participant characteristics; number (female %); age (years), mean (SD) or range	Reference method	No. of times the app was used	Dietary variables used for analysis	Statistical tests: Paired t-test/ Mann-Whitney U/Wilcoxon signed-rank test	Statistical tests: Median (range) of correlation coefficients: Pearson's (P), Spearman's (S)	Statistical tests: Bland–Altman analysis
Teixeira, V (2018) Brazil	University students 30 (73%); Age: 22.8 (2.6)	Two paper-based food records with Brazilian FCT	2 (on the same day of the food record)	Energy and 4 nutrients	(Mean) Energy: no difference<br><br>Nutrient (energy-adjusted): 3 significantly underestimated by the app	Energy S (crude): 0.70<br><br>Nutrient S (crude): 0.58 (0.53-0.59) S (energy-adjusted): 0.54 (0.53-0.63)	All: tendency for underestimation and relatively narrow limits of agreement. Energy, carbohydrate, and lipids: trends of increasing the degree of overestimation with increased intake
Chen, J (2019) Australia	Adults 43 (female % not indicated) Age: 32 (14)	Two 24-h dietary recalls with AUSNUT	4 (periods overlapping with the 24-h dietary recalls)	Energy and 4 nutrients	(Mean) Energy: significantly underestimated by the app<br><br>Nutrient (energy-adjusted): all significantly underestimated by the app	Energy P (crude): 0.25<br><br>Nutrient S or P(crude): 0.31 (0.21-0.42)	All: no proportional bias was observed; wide limits of agreement

AUSNUT, the Australian Food, Supplement, and Nutrient Database; FCT, food composition database; SD, standard deviation.

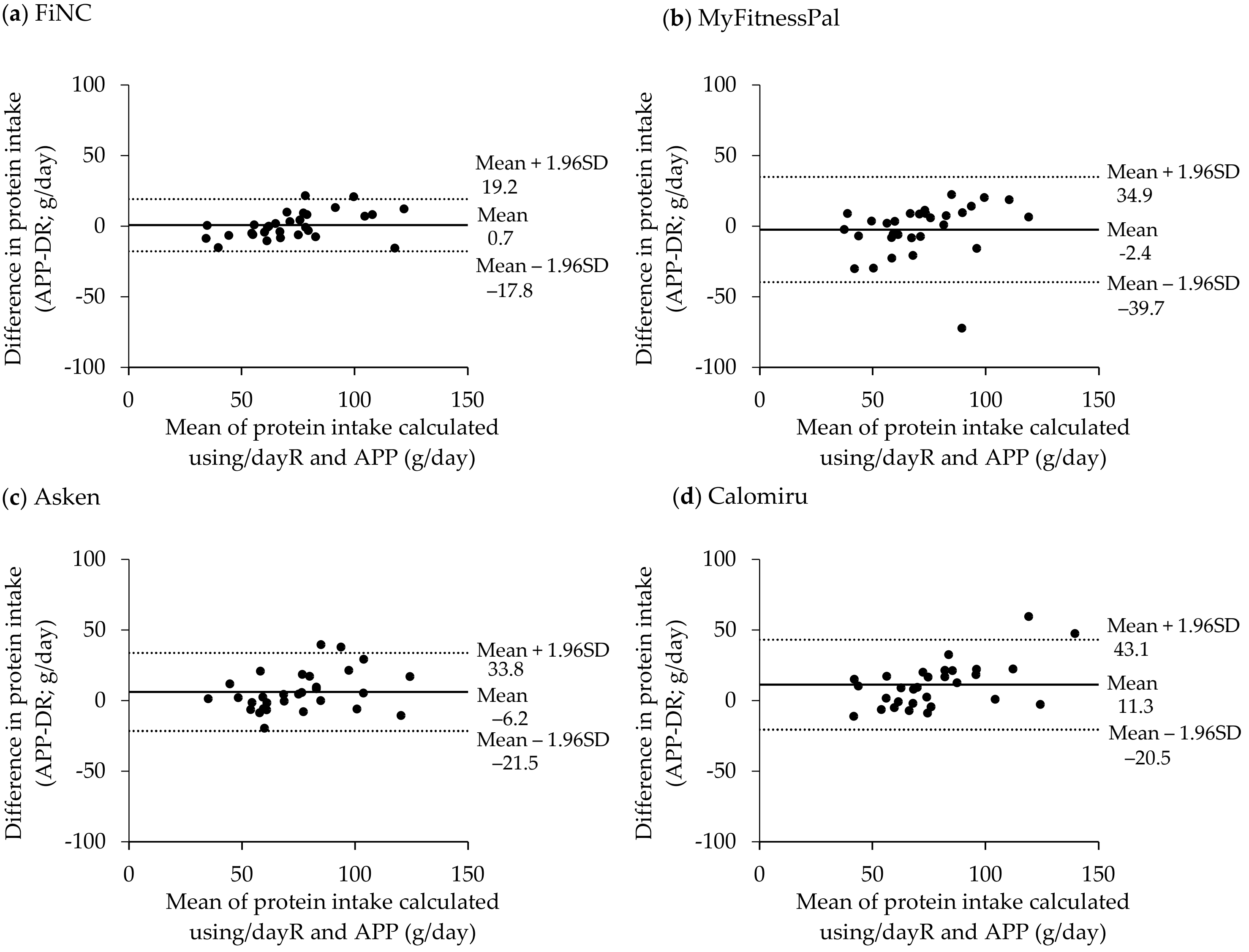

Figure S1. Bland–Altman plots assessing the agreement of the estimated protein intake between a paper-based dietary record (DR) and each application (APP) in 30 Japanese adults: (a) FiNC, (b) MyFitnessPal, (c) Asken, (d) Calomiru, and (e) Mogutan. The solid line represents the mean difference, and the dotted line represents lower and upper 95% limits of agreement.

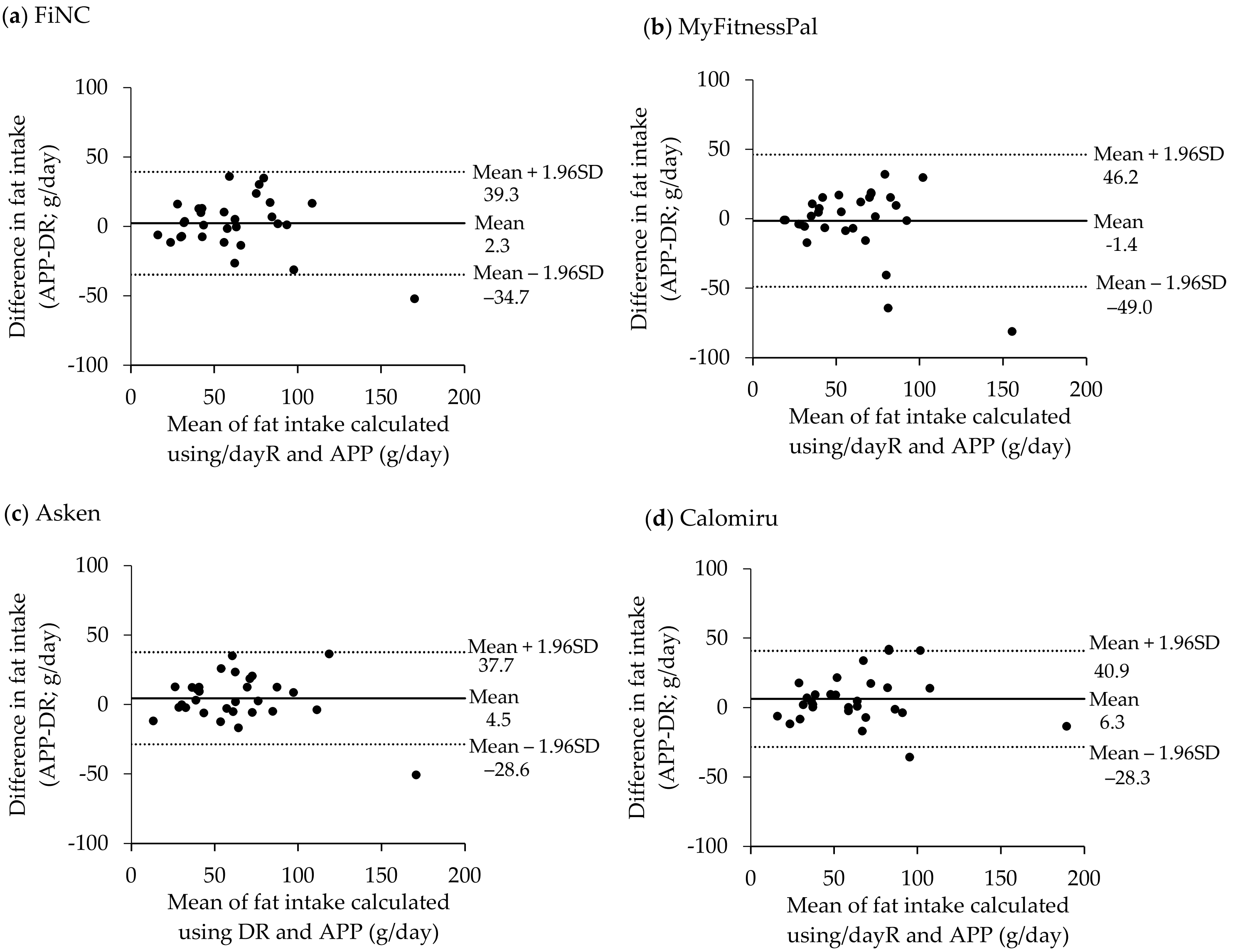

Figure S2. Bland–Altman plots assessing the agreement of the estimated fat intake between a paper-based dietary record (DR) and each application (APP) in 30 Japanese adults: (a) FiNC, (b) MyFitnessPal, (c) Asken, (d) Calomiru, and (e) Mogutan. The solid line represents the mean difference, and the dotted line represents lower and upper 95% limits of agreement.

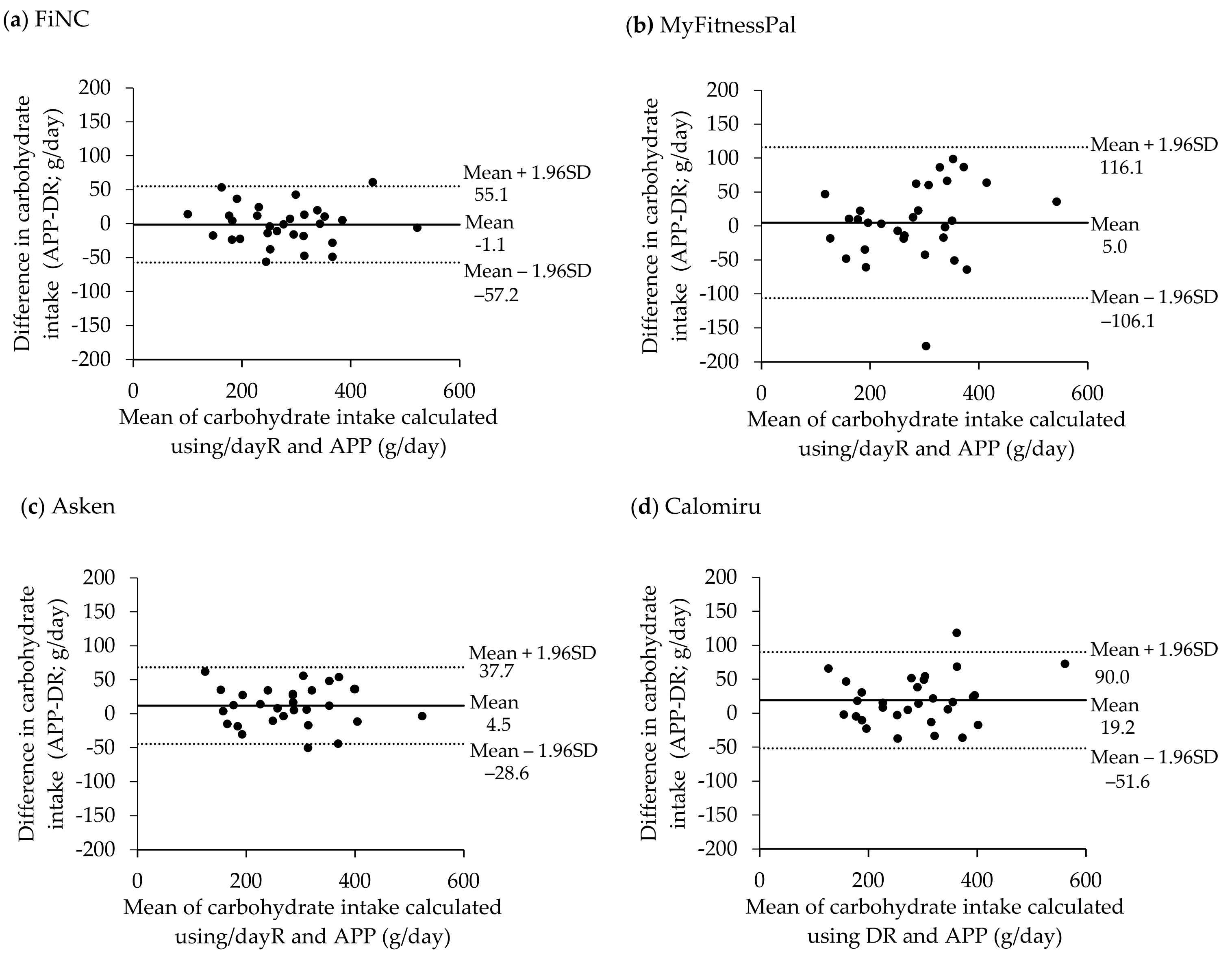

Figure S3. Bland–Altman plots assessing the agreement of the estimated carbohydrate intake between a paper-based dietary record (DR) and each application (APP) in 30 Japanese adults: (a) FiNC, (b) MyFitnessPal, (c) Asken, (d) Calomiru, and (e) Mogutan. The solid line represents the mean difference, and the dotted line represents lower and upper 95% limits of agreement.
